# Supplementary material for: Causal Relationship between Aging and Anorexia Nervosa: A White-Matter-Microstructure-Mediated Mendelian Randomization Analysis
Source: Biomedicines. 2024 Aug 16;12(8):1874. doi: 10.3390/biomedicines12081874 (PMC11351342; doi:10.3390/biomedicines12081874)
Supplement: Supplementary file 1 [file biomedicines-12-01874-s001.zip › Supplementary Figure/Figure S4/Singe SNP plot of MR analysis for TL to ubm-b-2030.pdf]

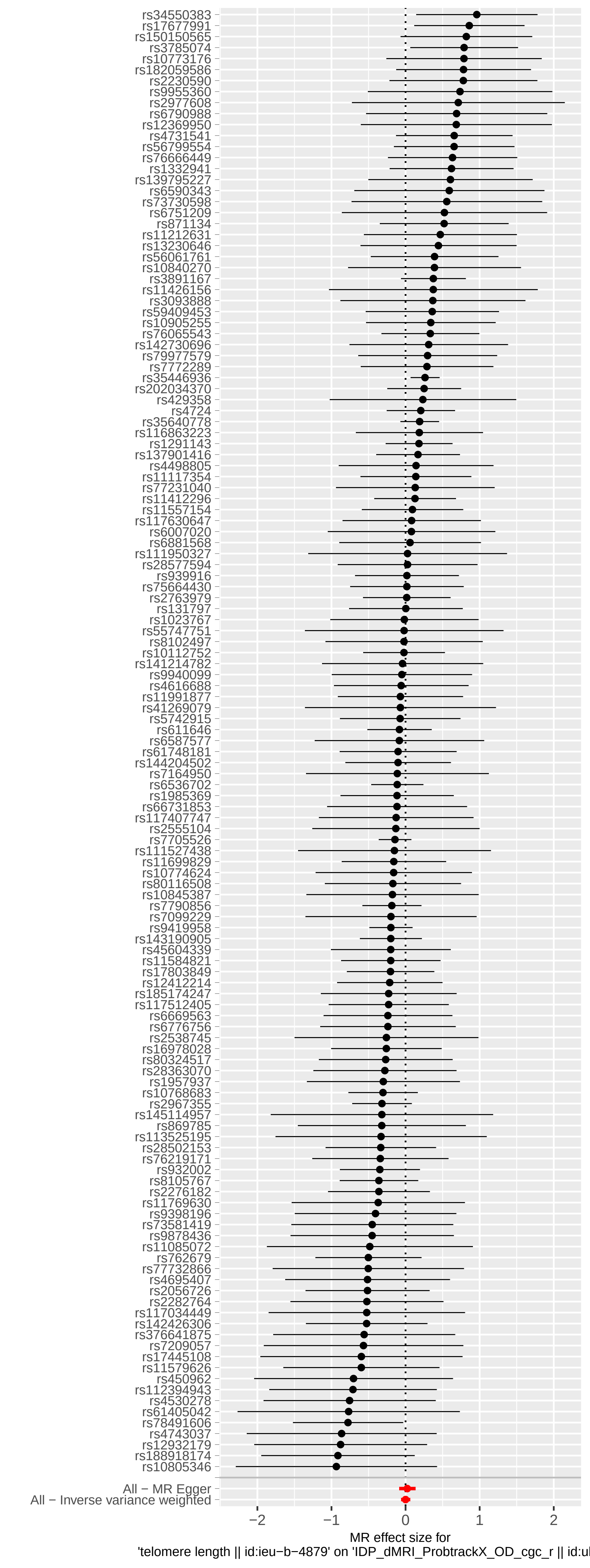

rs34550383
rs17677991
rs150150565
rs3785074
rs10773176
rs182059586
rs2230590
rs9955360
rs2977608
rs6790988
rs12369950
rs4731541
rs56799554
rs76666449
rs1332941
rs139795227
rs6590343
rs73730598
rs6751209
rs871134
rs11212631
rs13230646
rs56061761
rs10840270
rs3891167
rs11426156
rs3093888
rs59409453
rs10905255
rs76065543
rs142730696
rs79977579
rs7772289
rs35446936
rs202034370
rs429358
rs4724
rs35640778
rs116863223
rs1291143
rs137901416
rs4498805
rs11117354
rs77231040
rs11412296
rs11557154
rs117630647
rs6007020
rs6881568
rs111950327
rs28577594
rs939916
rs75664430
rs2763979
rs131797
rs1023767
rs55747751
rs8102497
rs10112752
rs141214782
rs9940099
rs4616688
rs11991877
rs41269079
rs5742915
rs611646
rs6587577
rs61748181
rs144204502
rs7164950
rs6536702
rs1985369
rs66731853
rs117407747
rs2555104
rs7705526
rs111527438
rs11699829
rs10774624
rs80116508
rs10845387
rs7790856
rs7099229
rs9419958
rs143190905
rs45604339
rs11584821
rs17803849
rs12412214
rs185174247
rs117512405
rs6669563
rs6776756
rs2538745
rs16978028
rs80324517
rs28363070
rs1957937
rs10768683
rs2967355
rs145114957
rs869785
rs113525195
rs28502153
rs76219171
rs932002
rs8105767
rs2276182
rs11769630
rs9398196
rs73581419
rs9878436
rs11085072
rs762679
rs77732866
rs4695407
rs2056726
rs2282764
rs117034449
rs142426306
rs376641875
rs7209057
rs17445108
rs11579626
rs450962
rs112394943
rs4530278
rs61405042
rs78491606
rs4743037
rs12932179
rs188918174
rs10805346
All − MR Egger
All − Inverse variance weighted
−2
−1
0
1
2
MR effect size for
'telomere length || id:ieu−b−4879' on 'IDP_dMRI_ProbtrackX_OD_cgc_r || id:ul
